# CRISPR/dCas9-Mediated DNA Methylation Editing on *emx2* in Chinese Tongue Sole (*Cynoglossus semilaevis*) Testis Cells

**DOI:** 10.3390/ijms25147637

**Published:** 2024-07-11

**Authors:** Yanxu Sun, Hong-Yan Wang, Binghua Liu, Bowen Yue, Qian Liu, Yuyan Liu, Ivana F. Rosa, Lucas B. Doretto, Shenglei Han, Lei Lin, Xiaoling Gong, Changwei Shao

**Affiliations:** 1 College of Fisheries and Life Science, Shanghai Ocean University, Shanghai 201306, China; 18854807173@163.com (Y.S.); 17852675181@163.com (B.Y.); xlgong@shou.edu.cn (X.G.); 2 State Key Laboratory of Mariculture Biobreeding and Sustainable Goods, Yellow Sea Fisheries Research Institute, Chinese Academy of Fishery Sciences, Qingdao 266071, China; wanghongyan@ysfri.ac.cn (H.-Y.W.); liubh@ysfri.ac.cn (B.L.); liuqian97927@163.com (Q.L.); liuyy@ysfri.ac.cn (Y.L.); lucas.doretto@unesp.br (L.B.D.); 17860712133@163.com (S.H.); linleicaas@163.com (L.L.); 3 Department of Structural and Functional Biology, Institute of Biosciences, São Paulo State University (UNESP), Botucatu 01049-010, Brazil; ivana.felipe@unesp.br; 4 Key Laboratory of Exploration and Utilization of Aquatic Genetic Resources (Shanghai Ocean University), Ministry of Education, Shanghai 201306, China; 5 National Demonstration Center for Experimental Fisheries Science Education, Shanghai Ocean University, Shanghai 201306, China; 6 Laboratory for Marine Fisheries Science and Food Production Processes, Qingdao Marine Science and Technology Center, Qingdao 266237, China

**Keywords:** CRISPR/dCas9, DNA methylation, *emx2*, *Cynoglossus semilaevis*

## Abstract

DNA methylation is a key epigenetic mechanism orchestrating gene expression networks in many biological processes. Nonetheless, studying the role of specific gene methylation events in fish faces challenges. In this study, we validate the regulation of DNA methylation on empty spiracles homeobox 2 (*emx2*) expression with decitabine treatment in Chinese tongue sole testis cells. We used the *emx2* gene as the target gene and developed a new DNA methylation editing system by fusing *dnmt3a* with catalytic dead Cas9 (dCas9) and demonstrated its ability for sequence-specific DNA methylation editing. Results revealed that utilizing dCas9-*dnmt3a* to target *emx2* promoter region led to increased DNA methylation levels and decreased *emx2* expression in Chinese tongue sole testis cells. More importantly, the DNA methylation editing significantly suppressed the expression of MYC proto-oncogene, bHLH transcription factor *(myc*), one target gene of *emx2*. Furthermore, we assessed the off-target effects of dCas9-*dnmt3a* and confirmed no significant impact on the predicted off-target gene expression. Taken together, we developed the first DNA methylation editing system in marine species and demonstrated its effective editing ability in Chinese tongue sole cells. This provides a new strategy for both epigenetic research and molecular breeding of marine species.

## 1. Introduction

Empty spiracles homeobox 2 (*EMX2*), a member of the Homeobox gene family, plays an important role as a developmental regulator in both growth and sex differentiation/determination [1]. In mice, *EMX2* transcripts are detectable in the “bipotential” gonad before the emergence of morphological differences between the sexes [2]. Specifically, gonadal regression occurs during the typical onset of sexual differentiation in both male and female knockout (KO) mice deficient in *EMX2* [3,4]. *EMX2* also regulates Wnt/β-catenin signaling [5], an important pathway for the development of gonads [6], while represses the expression of stem cell regulatory genes, such as *KLF4* and *MYC* [7]. In the marine fish Chinese tongue sole (*Cynoglossus semilaevis*), *emx2* is considered a potential target of gonadal DNA methylomes [8]. Variations across pseudomale (The genotype shows ZW, but the gonads develop into testis), female, and male fish DNA methylomes of *emx2* have showed an opposite trend to the gene expression levels. It suggests that the expression of *emx2* may be affected by DNA methylation. However, the precise regulatory mechanism of DNA methylation on *emx2* during sex determination and differentiation remains unclear. 

Recent studies involving DNA methyltransferase (DNMT) mutations have confirmed that changes in global DNA methylation levels can lead to male sterility in mice, highlighting its importance for normal spermatogenesis [9]. In mammals, a targeted addition of 5mC (5-methylcytosine, a common epigenetic modification in DNA) has been achieved by using human DNMT3A catalytic domain (DNMT3Acd) [10,11,12]. Moreover, 5-Aza-2′-deoxycytidine (5-Aza-dC), a DNA methyltransferase inhibitor employed to induce hypomethylation of the epigenome [13], and widely used in tissue culture and in animal models to facilitate promoter demethylation and reactivate gene expression was used. [14,15].These regulatory mechanisms may induce widespread global changes in methylation levels throughout the entire genome, leading to challenges in achieving methylation sequence-specific methylation modifications.

In recent years, the development of CRISPR/Cas9 has provided a new strategy for manipulating sequence-specific methylation. A combination of catalytically dead Cas9 nuclease (dCas9) and DNA methyltransferase has been employed to enhance sequence-specific methylation, while demethylase is used for DNA demethylation [16]. This system has demonstrated promise in gene function research and disease models, such as targeting the promoter of amyloid precursor protein (APP) via dCas9-Dnmt3a treatment to alter its DNA methylation for Alzheimer’s disease (AD) [11] and removing the 5mC target at the *FLOWERING WAGENINGEN* (*FWA*) promoter in the genome of *Arabidopsis* [17]. In this context, these studies suggest that the epigenetic editing system based on dCas9 is a powerful tool for DNA methylation studies and has broad research applications. Nevertheless, while previous studies predominately focused on human and model species, limited attention has been given to marine species.

In this study, we aimed to construct a system for single-gene targeted DNA methylation editing in Chinese tongue sole. For this purpose, we validate the regulatory role of DNA methylation on empty spiracles homeobox 2 (*emx2*) expression with decitabine treatment in Chinese tongue sole testis cells. And then, we developed a novel DNA methylation editing system by fusing *dnmt3a* with catalytically dead Cas9 (dCas9) and demonstrated its capability for sequence-specific DNA methylation editing. To the best of our knowledge, we developed the first DNA methylation editing system in marine species and demonstrated its effective editing ability in Chinese tongue sole cells. This approach offers a new strategy for advancing both epigenetic research and molecular breeding in marine species.

## 2. Results

### 2.1. Effects of 5-Aza-dC on emx2 Methylation and Expression

**Figure 1:**
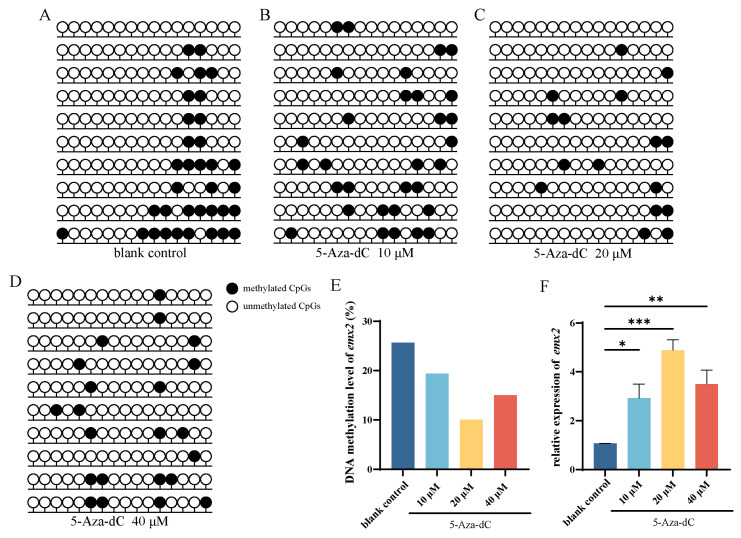
Effects of 5-Aza-dC-mediated demethylation on the *emx2* promoter followed by qPCR gene expression. CpGs’ methylation number was assessed for the (**A**) blank control and at three different concentrations of 5-Aza-dC treatment: (**B**) 10 μM, (**C**) 20 μM, and (**D**) 40 μM. (**E**) Percentage of DNA methylation levels at *emx2* promoter region with different 5-Aza-dC concentrations. (**F**) Relative expression of *emx2* with different concentrations of 5-Aza-dC. Filled (black) circles correspond to methylated CpGs; unfilled (white) circles correspond to unmethylated CpGs. Upper black lines indicate statistical differences among groups, while lines below the graph highlight the treated group compared to the control. The asterisks (*) are used to denote significant differences between groups using two-way ANOVA and Tukey’s multiple comparisons. * *p* < 0.05, ** *p* < 0.01, *** *p* < 0.001.

To investigate the connection between *emx2* gene expression and DNA methylation, we exposed dissociated testis cells from Chinese tongue sole to different concentrations of 5-Aza-dC. Bisulfite sequencing revealed a reduction in the number of methylated CpGs in the promoter region of *emx2* (Figure 1A–D, Figure S1) following 5-Aza-dC treatment. Bisulfite sequencing PCR (BSP) showed that DNA methylation of the non-treated cell group was approximately 25.63% (Figure 1E). A decrease in DNA methylation levels was evident in the *emx2* promoter region of the 5-Aza-dC-treated groups compared to the control group (Figure 1E). Particularly, treatment with 20 µM of 5-Aza-dC showed the most substantial reduction, with methylation levels of 10.63% (Figure 1E).

To clarify how the DNA methylation regulates gene expression of *emx2*, we used qPCR to confirm changes in gene expression following 5-Aza-dC treatment. The results showed an elevated expression of *emx2* after 48 h of treatment with different concentrations of 5-Aza-dC. The concentration 20 μM showed the most significantly increase (Figure 1F). 

### 2.2. Construction of CRISPR/dCas9-dnmt3a System in Chinese Tongue Sole

In order to make the building of the DNA methylation editing system so that it can be applied to the Chinese tongue sole, we cloned and analyzed the *dnmt3a* of Chinese tongue sole as the effector of editing system. We performed multiple sequence alignment analysis and phylogenetic tree construction for *dnmt3a* and conducted a comparative analysis between *dnmt3a* of Chinese tongue sole and other species, while all proteins contain conserved PWWP and ADDz domains and differ in their catalytic domains (Figures S2–S4). 

**Figure 2:**
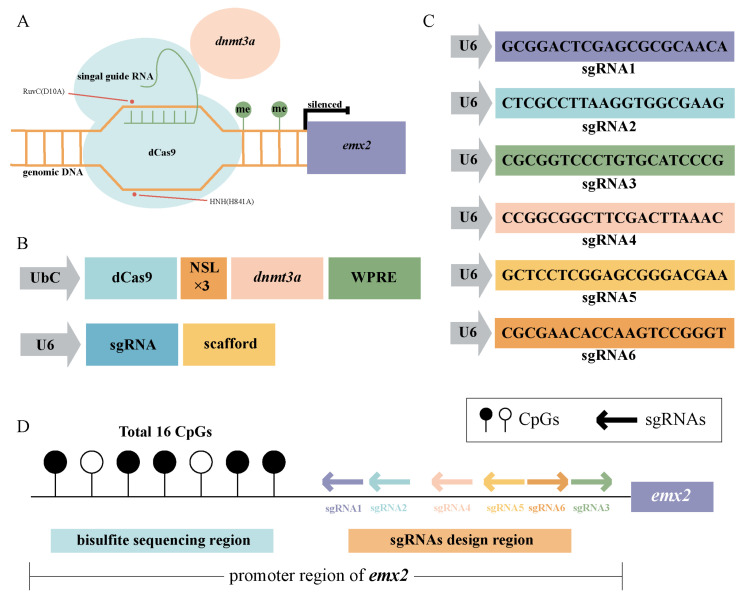
Schematic overview of the CRISPR/dCas9-*dnmt3a* editing system for the *emx2* promoter region of Chinese tongue sole testis cells. (**A**) Edit system schematic diagram. (**B**) dCas9-*dnmt3a* plasmid structure diagram and sgRNA plasmid structure diagram. (**C**) The sequence of the sgRNAs. (**D**) Bisulfite sequencing positions and sgRNA sites of *emx2*; lollipop chart on the left represents bisulfite sequencing of the CpGs’ position. The arrows indicate the target sequence of the designed sgRNAs, with the direction of the arrows aligning with the target sequence.

To construct a flexible system for targeting *emx2*, we used *dnmt3a* of Chinese tongue sole as an effector enzyme, fused with dCas9 protein. This construct was guided to the *emx2* promoter region using small guide RNAs (Figure 2A). The coding sequence (CDS) of *dnmt3a* was cloned and fused with dCas9 under the control of the constitutive ubiquitin C (UbC) promoter. Additionally, nuclear localization signals (NLS) were incorporated into both dCas9 and *dnmt3a* [12] (Figure 2B, Figure S5). Furthermore, we designed six sgRNAs targeting *emx2*, with each sgRNA driven by an independent U6 promoter (Figure 2B,C; Figure S6). Then, we designed a pair of methylation-specific PCR primers for the CpG Island of the *emx2* gene to amplify this DNA sequence. The position and the number of CpGs contained are shown in Figure 2D.

### 2.3. Targeted Methylation of the emx2 Promoter Region by dCas9-dnmt3a System

**Figure 3:**
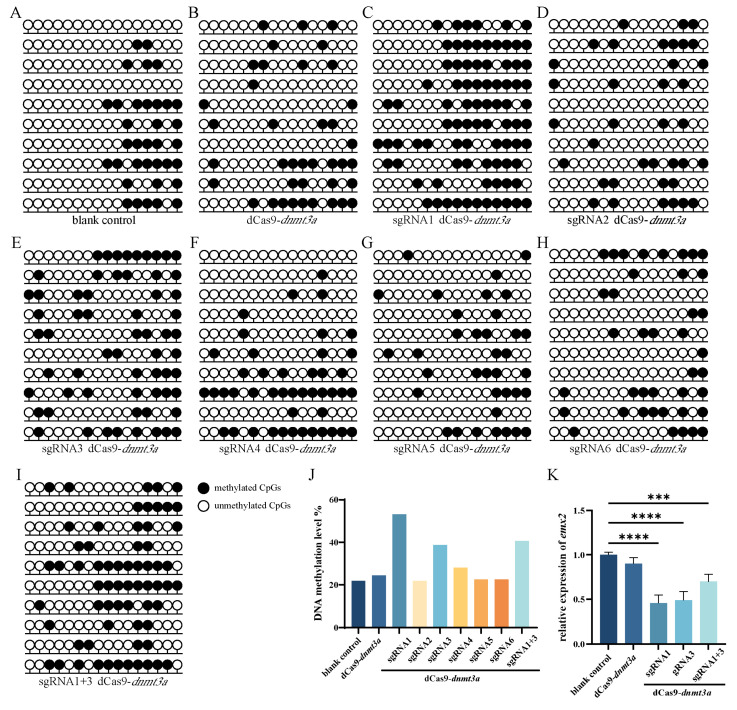
CRISPR/dCas9-mediated DNA methylation editing on *emx2* gene. DNA methylation status of the *emx2* promoter region with the (**A**) blank control; (**B**) dCas9-*dnmt3a*; (**C**) dCas9-*dnmt3a* and sgRNA1; (**D**) dCas9-*dnmt3a* and sgRNA2; (**E**) dCas9-*dnmt3a* and sgRNA3; (**F**) dCas9-*dnmt3a* and sgRNA4; (**G**) dCas9-*dnmt3a* and sgRNA5; (**H**) dCas9-*dnmt3a* and sgRNA6; (**I**) dCas9-*dnmt3a* and sgRNA1+3; (**J**) Percentage of DNA methylation levels at *emx2* promoter region with different treatments; (**K**) Relative expression of *emx2* after transfection of dCas9-*dnmt3a* and different sgRNAs. Filled (black) circles correspond to methylated CpGs, unfilled (white) circles correspond to unmethylated CpGs. Upper black lines indicate statistical difference among blank control and sgRNA groups, while lines below the graph highlight that the sgRNAs were co-transfected with the CRISPR/dCas9-*dnmt3a* vector. The asterisks (*) are used to denote significant differences among groups using two-way ANOVA and Tukey’s multiple comparisons. *** *p* < 0.001, **** *p* < 0.0001.

In order to investigate whether the dCas9-*dnmt3a* system can change the methylation level of the *emx2* promoter region, we transfected dCas9-*dnmt3a* plasmid with the sgRNA vector in Chinese tongue sole testis cells. Results of fluorescence intensity and relative expression of *dnmt3a* showed high transfection efficiency (Figure S7). Results of BSP showed that *emx2* methylation status differs after sgRNA-guided DNA methylation editing (Figure 3A–J). Among them, transfection with dCas9-*dnmt3a*/sgRNA1 and dCas9-*dnmt3a*/sgRNA3 resulted in a significant increase in DNA methylation in the *emx2* promoter region, rising about 30% and 15%, respectively (Figure 3J). However, other sgRNAs guiding DNA methylation editing showed no significant change (Figure 3D,F–H). Additionally, we tested whether dCas-*dnmt3a*/sgRNA1+3 had a better methylation effect. The BSP results indicate that the combined methylation effect of sgRNA1+sgRNA3 was similar to that of sgRNA1 and sgRNA3 individually, approximately 17.5% (Figure 3I,J). In the subsequent experiments, we targeted Chinese tongue sole cells with sgRNA1, sgRNA3 and sgRNA1+3. The qPCR analysis detected that *emx2* expression was dramatically decreased in sgRNAs’ guiding compared with the blank control and dCas9-*dnmt3a* with non-sgRNAs (Figure 3K), suggesting a successful addition of methylation at the *emx2* promoter and, consequently, resulting in significantly reduced expression of *emx2*.

### 2.4. Off-Target Effects of CRISPR/dCas9-dnmt3a System

**Figure 4:**
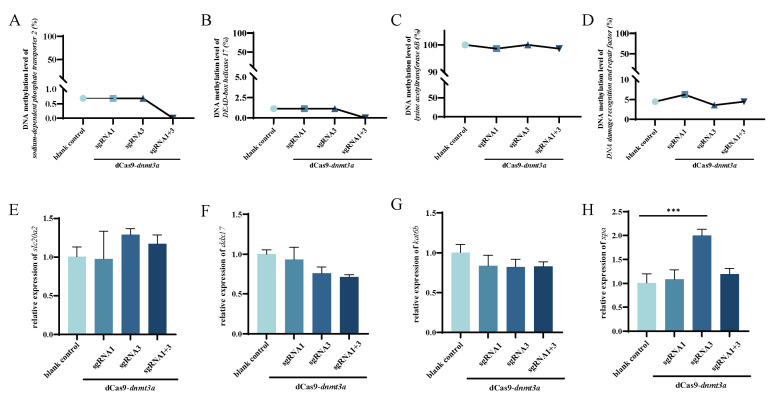
Off-target effects of CRISPR/dCas9-*dnmt3a* system. DNA methylation of the promoter region of (**A**) *slc20a2*; (**B**) *ddx17*; (**C**) *katb6* and (**D**) *xpa* and relative expression of potential off-target genes (**E**) *slc20a2*; (**F**) *ddx17*; (**G**) *katb6* and (**H**) *xpa*. Upper black lines indicate statistical difference between the blank control and sgRNA3 groups, while lines below the graph highlight that the sgRNAs were co-transfected with the CRISPR/dCas9-*dnmt3a* vector. The asterisks (*) are used to denote significant differences between groups using two-way ANOVA and Tukey’s multiple comparisons. *** *p* < 0.001.

To assess potential off-target effects, we analyzed four predicted off-target genes in the Chinese tongue sole genome using the sgRNAs’ sequence (sgRNA1 and sgRNA3) and the PAM sequence of Cas9 (NGG): sodium-dependent phosphate transporter 2 (*slc20a2*), DNA damage recognition and repair factor (*xpa*), DEAD-box helicase 17 (*ddx17*), and lysine acetyltransferase 6B (*kat6b*). BSP sequencing from the promoter of these genes indicated no significant change between the control and treatment groups (Figure 4A–D and Figure S8), except for a notable upregulation in the expression of *xpa* with sgRNA3 treatment (Figure 4E–H). However, since no methylation levels were altered in the *xpa* promoter region (Figure 4D), we do not attribute such change in expression to DNA methylation levels.

### 2.5. Methylation Editing on emx2 Affects Expression of Growth-Related Gene

**Figure 5:**
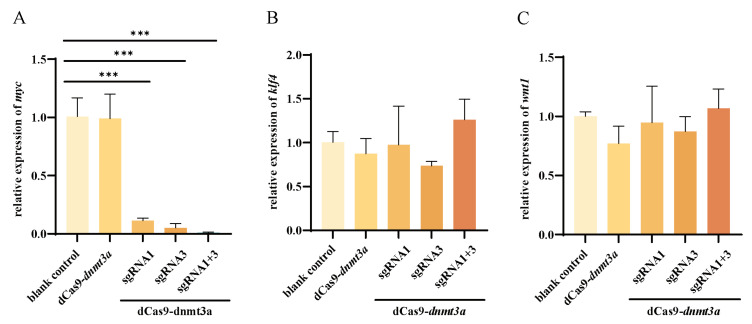
Effects on downstream genes after the *emx2* promoter region methylated. Relative expression of downstream genes (**A**) *myc*, (**B**) *klf4*, and (**C**) *wnt1*. Upper black lines indicate statistical difference among blank control and sgRNA groups for *myc* expression, while lines below the graph highlight that the sgRNAs were co-transfected with the CRISPR/dCas9-*dnmt3a* vector. The asterisks (*) are used to denote significant differences between groups using two-way ANOVA and Tukey’s multiple comparisons. *** *p* < 0.001.

Furthermore, we examined the changes in the regulation of downstream genes implicated in gonadal development and the maintenance of stem cell pluripotency after modulating *emx2* gene expression with the CRISPR/dCas9-*dnmt3a* system plus sgRNAs (Figure 5A–C). Results showed that qPCR analysis of wingless-type MMTV integration site family, member 1 (*wnt1*), Kruppel-like factor 4 (*klf4*), and *myc* expression showed that *myc* was downregulated in the groups where the sgRNAs were co-transfected with the CRISPR/dCas9-*dnmt3a* vector (Figure 5A), while the expression of *klf4* and *wnt1* was not significantly affected (Figure 5B,C).

## 3. Discussion

CRISPR/dCas9-based epigenetic editing technology has been widely used in mammals and plants due to its powerful editing and precise targeting ability combined with low off-target effects [17,18,19,20]. The use of this tool provides new strategies for controlling the occurrence of diseases and in animal growth studies [21,22]. Nonetheless, while previous studies predominately focused on human and mammals model species, limited attention has been given to marine species. Chinese tongue sole is considered a promising species for Chinese aquaculture due to its large size and high-quality meat [23], holding great potential to investigate gene regulation and inheritance mechanisms related to epigenetic mechanisms [8]. *emx2* is recognized for regulating embryonic development as well as for modulating transcriptional factors in different biological pathways [24,25,26] and epigenetic modification in fish sexual reversal [8]. Nonetheless, its regulation mechanisms involving DNA methylation remain largely unclear in fish gonads.

In our study, the relationship between *emx2* expression and DNA methylation was determined by treatment with 5-Aza-dC. This is a relatively common reagent to study the relationship between gene methylation level and expression. We are to build an *emx2* DNA methylation editing system in the process of finding that, in other species, the effector enzyme that performs DNA methylation editing, *dnmt3a*, is commonly of human origin. However, due to the differences in the catalytic domain of *dnmt3a* in different species, the use of human DNMT3Acd as an effector was unable to function in our system. So, we cloned the *dnmt3a* of Chinese tongue sole as the effector (Figures S2–S4). By fusing the *dnmt3a* gene of Chinese tongue sole to dCas9 to form a DNA methylation editing system (Figure 2), we targeted the *emx2* promoter region to directly change its DNA methylation levels. In addition, the potential off-target effects and the impact of editing on *emx2* downstream genes were also analyzed.

In this context, our initial findings indicated that the DNA methylation levels in cells derived from Chinese tongue sole testis were approximately 25.63% in the promoter region of *emx2* of the control group (Figure 1A). Following treatment with 5-Aza-Dc, the DNA methylation levels in this region decreased to approximately 10.63% (Figure 1E), accompanied by an upregulation of *emx2* expression across all concentrations (Figure 1F). As previously described in the literature, DNA methylation plays a crucial role in modulating the chromatin structure of regulatory regions in various genes, leading to a reduction in their expression levels [27,28]. Interestingly, the concentration of 20 μM 5-Aza-dC induced the most significant demethylation in the promoter region of *emx2*, resulting in the highest increase in transcript expression of this gene. 

Additionally, we successfully demonstrated that our DNA methylation editing system co-transfected with specific guides for Chinese tongue sole primary testis cells. Primary cells have a short time in vitro and do not undergo an immortalization process, so their biological characteristics have not changed much, and they still maintain the original genetic characteristics, which can better reflect the growth state of cells in vivo, so as to obtain data closer to the physiological function in vivo. In both the study by Michael Chavez and the study by Kanut Laoharawee, primary cells were used for CRISPR-based gene editing [29,30]. Thus, primary testis cells of Chinese tongue sole are of great significance for the study of targeted DNA methylation. After DNA methylation editing of *emx2*, we found that the sgRNA1, sgRNA3, and their combination sgRNA1/3, led to an increased methylation level compared to the control and dCas9-*dnmt3a* alone (Figure 3J), followed by a significant decrease in *emx2* expression (Figure 3K). The results showed that the DNA methylation level of CpG islands in the *emx2* promoter region was increased about 30% by the sgRNA1-guided editing system, about 15% by the sgRNA3-guided editing system, and about 17.5% by multiplexing of several sgRNA-guided editing systems. This indicated that the sgRNA3-guided editing system had a higher editing efficiency. The single gRNA leads to efficient and widespread methylation of the promoters. This conclusion is consistent with the work of Peter Stepper et al. on the editing efficiency of DNA methylation editing systems [10]. This expression pattern of *emx2* strongly suggests the success of our editing system in methylating the promoter region of *emx2*. To our knowledge, this study presents the first DNA methylation editing system in marine fish cells through an in vitro approach, which holds significant importance in various applications by elucidating the functional importance of DNA methylation in gene repression and controlling cellular differentiation status.

Given the widespread implementation of epigenetic editing, it is crucial to assess the specificity of chromatin editors, especially with CRISPR technologies, considering their potential to induce off-target effects [31]. To eliminate potential aberrant chromatin modifications at unrelated genomic loci, we screened the four most efficient potential off-target genes in the genome of Chinese tongue sole. These genes are based on Vanja Tadić et al.’s study about dCas9-based tools of off-target efficiency of the review, in accordance with the factors causing off-target to screen [32]. Our results show a conserved percentual methylation level at the promoter region of transfected cells compared to the control (Figure 4A–D) for all three guides. Despite the elevated levels of CpG methylation at untargeted sites throughout the genome of different species with dCas-fused DNMT3A reported in the literature [33], our results with the species-specific dCas9-*dnmt3a*/sgRNA system show low to no occurrence of off-target methylation, as also reported in some studies [34]. Furthermore, we also measured the expression levels of these genes to corroborate our off-target findings. Except for *xpa* expression following dCas9-*dnmt3a*/sgRNAs3 transfection (Figure 4H), no other gene modulation was observed (Figure 4A–G), confirming the high specificity of our dCas9-*dnmt3a*/sgRNAs3 system. Since no methylation levels were altered in the *xpa* promoter region (Figure 4D), we suggest that the downregulation of *myc* expression is not directly mediated by changes in DNA methylation levels within the *xpa* promoter region. These findings imply the existence of alternative regulatory mechanisms governing the relationship between *emx2* and *myc* expression. Further investigation is warranted to elucidate these alternative regulatory pathways, which may involve transcription factors, chromatin remodeling complexes, or other epigenetic modifications.

In this study, we also investigated the influence of dCas-fused *dnmt3a*/sgRNAs’ epigenetic effects in downstream genes and pathways related to Chinese tongue sole sexual reversal. Our findings indicated that the expression levels of *klf4* and *wnt1* remained stable following the methylation of the promoter region of *emx2*. In contrast, a notable downregulation of *myc* expression was observed (Figure 5). As the target gene of *emx2*, the altered expression of *myc* further verified the effectiveness of DNA methylation editing of the *emx2* gene. In addition, after editing for DNA methylation, testis cells’ mortality rate significantly increased (~30%) in the editing group, blank control group, and dCas9-*dnmt3a* with the non-sgRNA group without changing the number of cells significantly. We speculate that this may be caused by *myc*-regulated pathways, which we will further explore in subsequent studies.

In the context of fish, the inheritability of epigenetic editing technology remains a crucial topic for disease treatment, quality enhancement, and ornamental purposes. Therefore, further studies are needed to improve the editing efficiency, allowing for the practical application of epigenetic editing technology in fish. Taken together, we developed the first DNA methylation editing system in marine species and demonstrated its effective editing ability in Chinese tongue sole cells. This provides a new strategy for both epigenetic research and molecular breeding of marine species.

## 4. Materials and Methods

### 4.1. Experimental Fish Preparation

One-year-old Chinese tongue sole were obtained from Qingdao, China, and acclimatized to laboratory conditions for 48 h. Genotypic females and males were distinguished using specific markers [35], and physiological sex was confirmed post-dissection. Only male samples were retained for experimentation. Tissue excision was performed under MS222 anesthesia to minimize discomfort, adhering to Institutional Animal Care and Use Committee (IACUC) regulations (Approval No. YSFRI-2024011).

### 4.2. Cell Culture

One part of the gonad tissue was acquired from the Chinese tongue sole of three individuals and immediately placed in Leibovit’s L-15 with L-glutamine (Solarbio, LA9510, Beijing, China). Others were placed in EP tubes, quickly frozen in liquid nitrogen, and then frozen for long-time storage at −80 °C until DNA and RNA extraction. The gonad tissues were minced into small pieces and washed three times using a serum-free medium to remove attached blood and other impurities in a biosafety cabinet. Subsequently, pieces of gonad tissues were digested with 0.25% trypsin-EDTA (Gibco™, 25200072, Thermo Fisher Scientific, Waltham, MA, USA). The degree of cell digestion was observed under a microscope. The complete medium was added to terminate the digestion. Cells were seeded in cell culture flasks and transferred to the 24 °C cell culture incubator. About one week later, the growing adherent cells were observed under a microscope. The cells were incubated at 24 °C and replenished with fresh medium every 3 days. After 10 days, the cells migrated and grew into confluent monolayers and were subcultured by 0.25% trypsin-EDTA at a 1:2 split ratio. Cells grew into a confluent monolayer again after 3 days [36]. We used semi-quantitative RT-PCR to determine the predominant cell types in monolayer cells. We examined the gonadal somatic cell marker genes (*foxl2*, *sox9*, and *wt1a*) and germ cell marker genes (*dmrt1* and *vasa*) [37,38,39,40,41], the RT-PCR results were confirmed in at least 3 batches of independent experiments, and representative results are shown (Figure S9). The cells were used for subsequent experiments after passage 5.

### 4.3. In Vitro Treatment with 5-Aza-dC

5-Azacitidine-2′-deoxycytidine (5-Aza-dC, Macklin^®^, C10749023, Shanghai, China) was dissolved in 100% dimethyl sulfoxide (DMSO) to create a stock concentration of 10^5^ μM, which was stored at 4 °C. The stock solutions were then diluted with culture medium to obtain concentrations of 10 μM, 20 μM, and 40 μM. Cells were seeded in 6-well plates and allowed to grow overnight in a growth medium. Subsequently, cells were treated with various concentrations of 5-Aza-dC for 2 days before being isolated for further DNA and RNA extraction.

### 4.4. Molecular Cloning of dnmt3a and CRISPR/dCas9 Vector Construction

RNA from 1-year-old Chinese tongue sole was extracted and quantitatively reverse-transcribed using the HiScript^®^ III 1st Strand cDNA Synthesis Kit (+gDNA wiper) (Vazyme #R312, Nanjing, China), following the instruction manual. The amplification primers were designed according to the *dnmt3a* X1 sequence of Chinese tongue sole from NCBI (Gene ID: 103380388) [42,43] (Table S1). The PCR cloning reactions were performed by using the high-fidelity enzymes PrimeSTAR^®^ Max DNA Polymerase (TAKARA, R045Q, Osaka, Japan). The reaction system of PCR was 50 µL, containing 25 µL of PrimeSTAR Max Premix (2×), 2 µL of cDNA, 1.5 µL of primer F, 1.5 µL of primer R, and 20 µL of DEPC water. The PCR program was as follows: 5 min at 95 °C for predenaturation, followed by 35 cycles of 95 °C for 15 s, 60 °C for 1 min, and 72 °C for 15 s. The PCR products were subjected to 1% agarose gel electrophoresis to separate them, and bands of the desired sizes were excised, purified, and ligated with the pEAZY-T1 vector for Sanger sequencing.

The *dnmt3a* sequence and the fuw-dCas9 plasmid (Addgene #84476, Watertown, MA, USA) were digested with HamI (NEB) and EcoRI (NEB) enzymes at 37 °C for 2 h, respectively. Subsequently, we used T4 Ligase (TAKARA) to form the final circular fuw-dCas9-*dnmt3a* plasmid. Plasmid extraction was performed using a TIANGEN plasmid extraction kit (TIANGEN, DP118, Beijing, China) without endotoxin, and the plasmid was stored at −20 °C.

### 4.5. The sgRNA Vectors’ Construction

The online tool MethPrimer (https://www.urogene.org/methprimer/, accessed on 10 April 2023) was used to analyze the size and locations of CpG islands of the *emx2* promoter region [44]. With the location of the CpG Island and TSS of *emx2*, six sgRNAs at different positions were designed by using the online website tool CRISPOR (http://crispor.gi.ucsc.edu/, accessed on 8 May 2023). We used a reverse PCR method to construct the designed forward and reverse primers into the plasmid pGL3-U6-sgRNA-PGK (Addgene plasmid # 51133). The reaction system of PCR was 20 µL, containing 10 µL of KDO (2 × Master Mix, TOYOBO, Osaka, Japan), 1 µL of sgRNA plasmid, 0.5 µL of primer F, 0.5 µL of primer R, and 8 µL of DEPC water. The PCR program was as follows: 5 min at 95 °C for predenaturation, followed by 35 cycles of 95 °C for 15 s, 50 °C for 1 min, and 72 °C for 15 s. Primer sequences are described in detail in Table S2. Plasmid was extracted with a TAKARA plasmid extraction kit without endotoxin (TIANGEN, DP118) and stored at −20 °C.

### 4.6. Cell Transfection

The cells were seeded in growth medium in 6-well plates and incubated overnight. Experiments were conducted when the cell density reached 80% and replenished with fresh medium with 50% serum reduced. To assess potential off-target effects of sgRNA-guided dCas9-*dnmt3a*, two control groups were established: a blank control and a dCas9-*dnmt3a* with a non-sgRNA control, each with three replicates. We used Lipofectamine™ 3000 (Thermo Fisher, Waltham, MA, USA) for transfection by liposome-mediated transfection. Each well was transfected with 1000 ng of both sgRNAs and dCas9-*dnmt3a* plasmids. We also transfected an additional 500 ng of plasmid expressing green fluorescent protein (GFP) to characterize transfection efficiency. Following transfection for approximately 6–8 h, the medium was reverted to full serum medium and cultured for three consecutive days, during which the growth status and fluorescence intensity of the cells were observed.

### 4.7. Total RNA and DNA Extraction

Half of the cells in each well were harvested by scraping with a cell scraper, and total cell DNA was extracted using the Vazyme FastPure Blood/Cell/Tissue/Bacteria DNA Isolation Mini Kit (Vazyme #dC112), following the manufacturer’s instructions. After extraction, the DNA was dissolved in sterile enzyme-free water, stored at −20 °C, and long-term-stored at −80 °C refrigerator. Trizol reagent (Invitrogen 15596026CN, Thermo Fisher, Waltham, MA, USA) was added at a volume of 500 μL to each well, and total RNA was extracted using the Trizol manufacturer’s protocol. The Trizol, chloroform, and isopropanol were mixed at a ratio of approximately 5:1:2. The resulting mixture was used for total RNA extraction. Subsequently, the extracted RNA was dissolved and diluted with water, and its concentration and quality were assessed. The RNA samples were then stored at −80 °C in the refrigerator.

### 4.8. RT-qPCR

A PrimeScript™ RT reagent Kit with gDNA Eraser (Perfect Real Time) was used for cDNA synthesis for reverse-transcriptase qPCR, following the instruction manual (Takara RR047A, Osaka, Japan). Primer sequences for qPCR were designed by NCBI Blast tool (Table S3). A 2 × QuantiFast SYBR Green RT-PCR Kit (Qiangen, Cat. No./ID: 204156, Shanghai, China) was used to amplify the target gene. Qualitative detection and melting curve analysis was performed by the LightCycler^®^ 96 Instrument (Roche LightCycler^®^ 96 Instrument, Basel, Switzerland), a precise 96-well real-time PCR device. The reaction system of qPCR was 20 µL, containing 10 µL of SYBR, 2 µL of cDNA, 1 µL of primer F, 1 µL of primer R, and 6 µL of DEPC water. Melting curves were used to determine amplification specificity. After the reaction, according to the internal genes (*beta-actin*) and Ct value of the target genes, the 2^−ΔΔ^Ct method was used to determine the relative expression level of target genes [45]. All experiments were set up with three biological and three technical replicates.

### 4.9. Bisulfite PCR Sequencing

DNA was treated with bisulfite using the Vazyme EpiArt DNA Methylation Bisulfite Kit (Vazyme EM101-01, Nanjing, China). Libraries were generated from purified PCR products amplified from the bisulfite-treated DNA by using primers designed from MethPrimer (MethPrimer (https://www.urogene.org/methprimer/, accessed on 10 April 2023)) (Table S4), using Vazyme 2×EpiArt HS Taq Master Mix (Dye Plus) (Vazyme EM202). PCR products were identified by agarose gel electrophoresis, and bands of corresponding size were cut, purified, and ligated with the Pesy-T1 vector for Sanger sequencing. The sequenced sequences were imported into BiQ-Analyzer software (https://biq-analyzer.bioinf.mpi-inf.mpg.de/, accessed on 10 January 2024) for methylation analysis [46]. Methylated CpGs are set in black and unmethylated CpGs in white.

### 4.10. Bioinformatic Analysis and Phylogenetic Tree Construction

DNAMAN was used to compare the sequencing sequence and the CDS sequence of *dnmt3a* X1 in the NCBI database, and the sequence information was analyzed. An NCBI online tool (NCBI Conserved Domain Search (https://www.ncbi.nlm.nih.gov/Structure/cdd/wrpsb.cgi, accessed on 3 March 2024)) was used to predict the *dnmt3a* domain of Chinese tongue sole with the help of NCBI’s Conserved Domain Database [47,48,49]. The amino acid sequences of closely related species of Chinese tongue sole and the model species at a key position in the evolution were selected for multiple sequence alignment and phylogenetic tree construction. Phylogenetic tree construction was performed using the IQ-TREE 2 (http://www.iqtree.org, last accessed on 6 February 2020) tool, and phylogenetic inference was performed using the maximum likelihood (ML) criterion. We ran 10,000 ultra-fast bootstrap (UFBoot) replicates [50]. Multiple sequence alignments were performed using the tool EMBL-EBI (https://www.ebi.ac.uk/, accessed on 3 March 2024) [51].

### 4.11. Statistical Analysis

Results were analyzed and plotted using GraphPad Prism (GraphPrism 9.0) and Microsoft Excel (Microsoft Office 2010) software. Each experiment was performed in triplicate. Statistical analysis among different groups was performed using two-way ANOVA and Tukey’s multiple comparisons. The data were presented as mean ± standard error of the mean (SEM), and *p* < 0.05 was considered significant. (*, *p* < 0.05; **, *p* < 0.01; ***, *p* < 0.001; ****, *p* < 0.0001).

## 5. Conclusions

In this study, we validated the regulation of DNA methylation on *emx2* expression with decitabine treatment in Chinese tongue sole testis cells. We developed a new DNA methylation editing system and demonstrated its ability for sequence-specific DNA methylation editing. It targets the *emx2* promoter region and led to increased DNA methylation levels and decreased *emx2* expression in Chinese tongue sole testis cells. DNA methylation editing significantly suppressed the expression of *myc*, one target gene of *emx2*. Furthermore, we assessed the off-target effects of dCas9-*dnmt3a* and confirmed no significant impact on the predicted off-target gene expression. Taken together, we developed the first DNA methylation editing system in marine species and demonstrated its effective editing ability in Chinese tongue sole cells. This provides a new strategy for both epigenetic research and molecular breeding of marine species. 

## 6. Patents

This manuscript describes inventions covered in the Chinese patent application No. 202410463573.1 (C.S. and Y.S., inventors).

## Data Availability

Data are contained within the article.

## Supplementary Materials

The following supporting information can be downloaded at: https://www.mdpi.com/article/10.3390/ijms25147637/s1.

## References

[1] Abate-Shen C. (2002). Deregulated homeobox gene expression in cancer: Cause or consequence?. Nat. Rev. Cancer.

[2] Pellegrini M., Pantano S., Lucchini F., Fumi M., Forabosco A. (1997). Emx2 developmental expression in the primordia of the reproductive and excretory systems. Anat. Embryol..

[3] Miyamoto N., Yoshida M., Kuratani S., Matsuo I., Aizawa S. (1997). Defects of urogenital development in mice lacking Emx2. Dev. (Camb. Engl.).

[4] Kusaka M., Katoh-Fukui Y., Ogawa H., Miyabayashi K., Baba T., Shima Y., Sugiyama N., Sugimoto Y., Okuno Y., Kodama R. (2010). Abnormal epithelial cell polarity and ectopic epidermal growth factor receptor (EGFR) expression induced in Emx2 KO embryonic gonads. Endocrinology.

[5] Jimenez-García M.P., Lucena-Cacace A., Otero-Albiol D., Carnero A. (2021). Empty spiracles homeobox genes EMX1 and EMX2 regulate WNT pathway activation in sarcomagenesis. J. Exp. Clin. Cancer Res. CR.

[6] van der Horst P.H., Wang Y., van der Zee M., Burger C.W., Blok L.J. (2012). Interaction between sex hormones and WNT/β-catenin signal transduction in endometrial physiology and disease. Mol. Cell. Endocrinol..

[7] Jimenez-García M.P., Lucena-Cacace A., Otero-Albiol D., Carnero A. (2021). Regulation of sarcomagenesis by the empty spiracles homeobox genes EMX1 and EMX2. Cell Death Dis..

[8] Shao C., Li Q., Chen S., Zhang P., Lian J., Hu Q., Sun B., Jin L., Liu S., Wang Z. (2014). Epigenetic modification and inheritance in sexual reversal of fish. Genome Res..

[9] Dura M., Teissandier A., Armand M., Barau J., Lapoujade C., Fouchet P., Bonneville L., Schulz M., Weber M., Baudrin L.G. (2022). DNMT3A-dependent DNA methylation is required for spermatogonial stem cells to commit to spermatogenesis. Nat. Genet..

[10] Stepper P., Kungulovski G., Jurkowska R.Z., Chandra T., Krueger F., Reinhardt R., Reik W., Jeltsch A., Jurkowski T.P. (2017). Efficient targeted DNA methylation with chimeric dCas9-Dnmt3a-Dnmt3L methyltransferase. Nucleic Acids Res..

[11] Park H., Shin J., Kim Y., Saito T., Saido T.C., Kim J. (2022). CRISPR/dCas9-Dnmt3a-mediated targeted DNA methylation of APP rescues brain pathology in a mouse model of Alzheimer’s disease. Transl. Neurodegener..

[12] Liu X.S., Wu H., Ji X., Stelzer Y., Wu X., Czauderna S., Shu J., Dadon D., Young R.A., Jaenisch R. (2016). Editing DNA Methylation in the Mammalian Genome. Cell.

[13] Seelan R.S., Mukhopadhyay P., Pisano M.M., Greene R.M. (2018). Effects of 5-Aza-2’-deoxycytidine (decitabine) on gene expression. Drug Metab. Rev..

[14] Bhat S.A., Sureshbabu S.K., Philip C.S., Chiplunkar S., Kabelitz D., Bhat J. (2020). Chapter 14—Impact of epigenetic modifiers on the immune system. Epigenetics of the Immune System.

[15] Liu S., Tan J., Wu C., Wang L. (2021). Chapter 2—DNA methyltransferase inhibitors (DNMTis) as sensitizing agents to overcome chemoresistance. Epigenetic Regulation in Overcoming Chemoresistance.

[16] Nunez J.K., Chen J., Pommier G.C., Cogan J.Z., Replogle J.M., Adriaens C., Ramadoss G.N., Shi Q., Hung K.L., Samelson A.J. (2021). Genome-wide programmable transcriptional memory by CRISPR-based epigenome editing. Cell.

[17] Gallego-Bartolomé J., Gardiner J., Liu W., Papikian A., Ghoshal B., Kuo H.Y., Zhao J.M., Segal D.J., Jacobsen S.E. (2018). Targeted DNA demethylation of the Arabidopsis genome using the human TET1 catalytic domain. Proc. Natl. Acad. Sci. USA.

[18] Vojta A., Dobrinić P., Tadić V., Bočkor L., Korać P., Julg B., Klasić M., Zoldoš V. (2016). Repurposing the CRISPR-Cas9 system for targeted DNA methylation. Nucleic Acids Res..

[19] Baumann V., Wiesbeck M., Breunig C.T., Braun J.M., Köferle A., Ninkovic J., Götz M., Stricker S.H. (2019). Targeted removal of epigenetic barriers during transcriptional reprogramming. Nat. Commun..

[20] Marx N., Grünwald-Gruber C., Bydlinski N., Dhiman H., Ngoc Nguyen L., Klanert G., Borth N. (2018). CRISPR-Based Targeted Epigenetic Editing Enables Gene Expression Modulation of the Silenced Beta-Galactoside Alpha-2,6-Sialyltransferase 1 in CHO Cells. Biotechnol. J..

[21] Nakamura M., Gao Y., Dominguez A.A., Qi L.S. (2021). CRISPR technologies for precise epigenome editing. Nat. Cell Biol..

[22] Mkannez G., Gagné-Ouellet V., Jalloul Nsaibia M., Boulanger M.C., Rosa M., Argaud D., Hadji F., Gaudreault N., Rhéaume G., Bouchard L. (2018). DNA methylation of a PLPP3 MIR transposon-based enhancer promotes an osteogenic programme in calcific aortic valve disease. Cardiovasc. Res..

[23] Valente L.M.P., Moutou K.A., Conceição L.E.C., Engrola S., Fernandes J.M.O., Johnston I.A. (2013). What determines growth potential and juvenile quality of farmed fish species?. Rev. Aquac..

[24] Aykut B., Ochs M., Radhakrishnan P., Brill A., Höcker H., Schwarz S., Weissinger D., Kehm R., Kulu Y., Ulrich A. (2017). EMX2 gene expression predicts liver metastasis and survival in colorectal cancer. BMC Cancer.

[25] Cecchi C., Boncinelli E. (2000). Emx homeogenes and mouse brain development. Trends Neurosci..

[26] Hatch K., Pabon A., DiMario J.X. (2017). EMX2 activates slow myosin heavy chain 2 gene expression in embryonic muscle fibers. Mech. Dev..

[27] Bird A.P., Wolffe A.P. (1999). Methylation-induced repression—Belts, braces, and chromatin. Cell.

[28] He L., Huang H., Bradai M., Zhao C., You Y., Ma J., Zhao L., Lozano-Durán R., Zhu J.K. (2022). DNA methylation-free Arabidopsis reveals crucial roles of DNA methylation in regulating gene expression and development. Nat. Commun..

[29] Chavez M., Rane D.A., Chen X., Qi L.S. (2023). Stable expression of large transgenes via the knock-in of an integrase-deficient lentivirus. Nat. Biomed. Eng..

[30] Laoharawee K., Johnson M.J., Moriarity B.S. (2020). CRISPR/Cas9-Mediated Genome Engineering of Primary Human B Cells. Methods Mol. Biol..

[31] Kim D., Luk K., Wolfe S.A., Kim J.S. (2019). Evaluating and Enhancing Target Specificity of Gene-Editing Nucleases and Deaminases. Annu. Rev. Biochem..

[32] Tadić V., Josipović G., Zoldoš V., Vojta A. (2019). CRISPR/Cas9-based epigenome editing: An overview of dCas9-based tools with special emphasis on off-target activity. Methods.

[33] Lin L., Liu Y., Xu F., Huang J., Daugaard T.F., Petersen T.S., Hansen B., Ye L., Zhou Q., Fang F. (2018). Genome-wide determination of on-target and off-target characteristics for RNA-guided DNA methylation by dCas9 methyltransferases. GigaScience.

[34] Yamazaki T., Hatano Y., Handa T., Kato S., Hoida K., Yamamura R., Fukuyama T., Uematsu T., Kobayashi N., Kimura H. (2017). Targeted DNA methylation in pericentromeres with genome editing-based artificial DNA methyltransferase. PLoS ONE.

[35] Wang X., Jiang J., Gao J., Liu J., Qi J., Wang Z., Yu H., Zhang Q. (2013). Identification of two novel female-specific DNA sequences in half-smooth tongue sole, *Cynoglossus semilaevis*. Aquaculture.

[36] Sun A., Chen S.L., Gao F.T., Li H.L., Liu X.F., Wang N., Sha Z.X. (2015). Establishment and characterization of a gonad cell line from half-smooth tongue sole *Cynoglossus semilaevis* pseudomale. Fish Physiol. Biochem..

[37] Yamaguchi T., Yamaguchi S., Hirai T., Kitano T. (2007). Follicle-stimulating hormone signaling and Foxl2 are involved in transcriptional regulation of aromatase gene during gonadal sex differentiation in Japanese flounder, Paralichthys olivaceus. Biochem. Biophys. Res. Commun..

[38] Ijiri S., Kaneko H., Kobayashi T., Wang D.S., Sakai F., Paul-Prasanth B., Nakamura M., Nagahama Y. (2008). Sexual dimorphic expression of genes in gonads during early differentiation of a teleost fish, the Nile tilapia Oreochromis niloticus. Biol. Reprod..

[39] Chiang E.F., Pai C.I., Wyatt M., Yan Y.L., Postlethwait J., Chung B. (2001). Two sox9 genes on duplicated zebrafish chromosomes: Expression of similar transcription activators in distinct sites. Dev. Biol..

[40] Guo Y., Cheng H., Huang X., Gao S., Yu H., Zhou R. (2005). Gene structure, multiple alternative splicing, and expression in gonads of zebrafish Dmrt1. Biochem. Biophys. Res. Commun..

[41] Kobayashi T., Kajiura-Kobayashi H., Nagahama Y. (2002). Two isoforms of vasa homologs in a teleost fish: Their differential expression during germ cell differentiation. Mech. Dev..

[42] Burge S., Kelly E., Lonsdale D., Mutowo-Muellenet P., McAnulla C., Mitchell A., Sangrador-Vegas A., Yong S.Y., Mulder N., Hunter S. (2012). Manual GO annotation of predictive protein signatures: The InterPro approach to GO curation. Database J. Biol. Databases Curation.

[43] Lu Y.F., Liu Q., Liu K.Q., Wang H.Y., Li C.H., Wang Q., Shao C.W. (2022). Identification of global alternative splicing and sex-specific splicing via comparative transcriptome analysis of gonads of Chinese tongue sole (*Cynoglossus semilaevis*). Zool. Res..

[44] Li L.C., Dahiya R. (2002). MethPrimer: Designing primers for methylation PCRs. Bioinformatics.

[45] Livak K.J., Schmittgen T.D. (2001). Analysis of relative gene expression data using real-time quantitative PCR and the 2(-Delta Delta C(T)) Method. Methods.

[46] Bock C., Reither S., Mikeska T., Paulsen M., Walter J., Lengauer T. (2005). BiQ Analyzer: Visualization and quality control for DNA methylation data from bisulfite sequencing. Bioinformatics.

[47] Lu S., Wang J., Chitsaz F., Derbyshire M.K., Geer R.C., Gonzales N.R., Gwadz M., Hurwitz D.I., Marchler G.H., Song J.S. (2020). CDD/SPARCLE: The conserved domain database in 2020. Nucleic Acids Res..

[48] Marchler-Bauer A., Bryant S.H. (2004). CD-Search: Protein domain annotations on the fly. Nucleic Acids Res..

[49] Marchler-Bauer A., Lu S., Anderson J.B., Chitsaz F., Derbyshire M.K., DeWeese-Scott C., Fong J.H., Geer L.Y., Geer R.C., Gonzales N.R. (2011). CDD: A Conserved Domain Database for the functional annotation of proteins. Nucleic Acids Res..

[50] Minh B.Q., Schmidt H.A., Chernomor O., Schrempf D., Woodhams M.D., von Haeseler A., Lanfear R. (2020). IQ-TREE 2: New Models and Efficient Methods for Phylogenetic Inference in the Genomic Era. Mol. Biol. Evol..

[51] Madeira F., Pearce M., Tivey A.R.N., Basutkar P., Lee J., Edbali O., Madhusoodanan N., Kolesnikov A., Lopez R. (2022). Search and sequence analysis tools services from EMBL-EBI in 2022. Nucleic Acids Res..

