# Supplementary material for: CRISPR/dCas9-Mediated DNA Methylation Editing on emx2 in Chinese Tongue Sole (Cynoglossus semilaevis) Testis Cells
Source: Int J Mol Sci. 2024 Jul 11;25(14):7637. doi: 10.3390/ijms25147637 (PMC11277268; doi:10.3390/ijms25147637)
Supplement: Supplementary file 1 [file ijms-25-07637-s001.zip › ijms-3054143-supplementary.pdf]

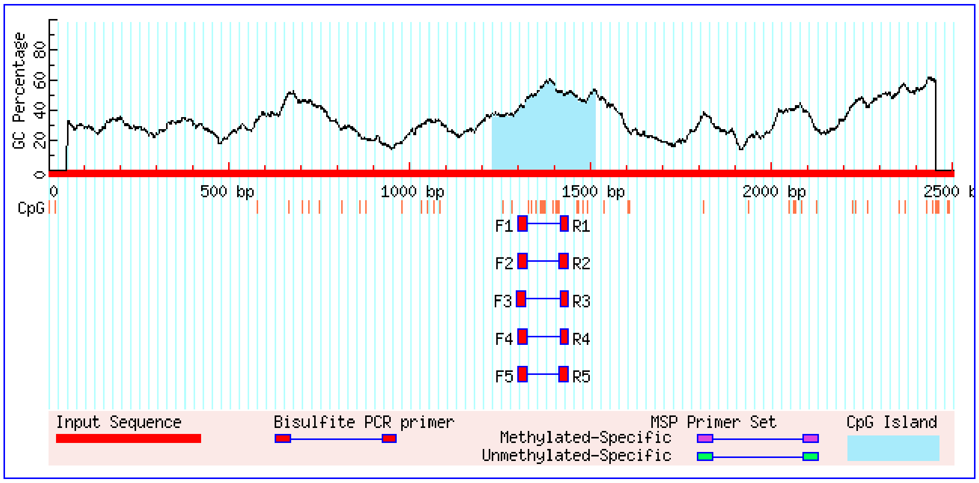

Figure S1. Predictive analytics of *emx2* promoter. The blue shading represents CpG island.

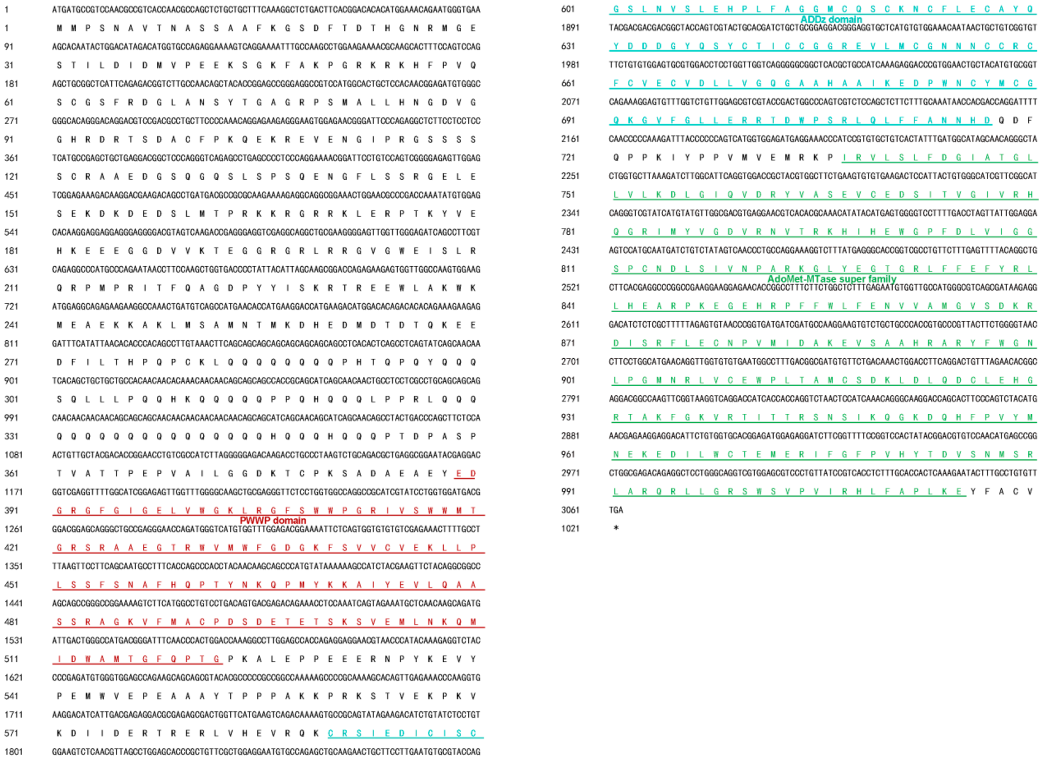

Figure S2. The cDNA sequencing and the acid sequence of *dnmt3a* from Chinese tongue sole. The underlined amino acid sequences respresent the three domains. Including one PWWP domain (red), one ADDz domian (blue) and one Adomet_MTase super family (green).

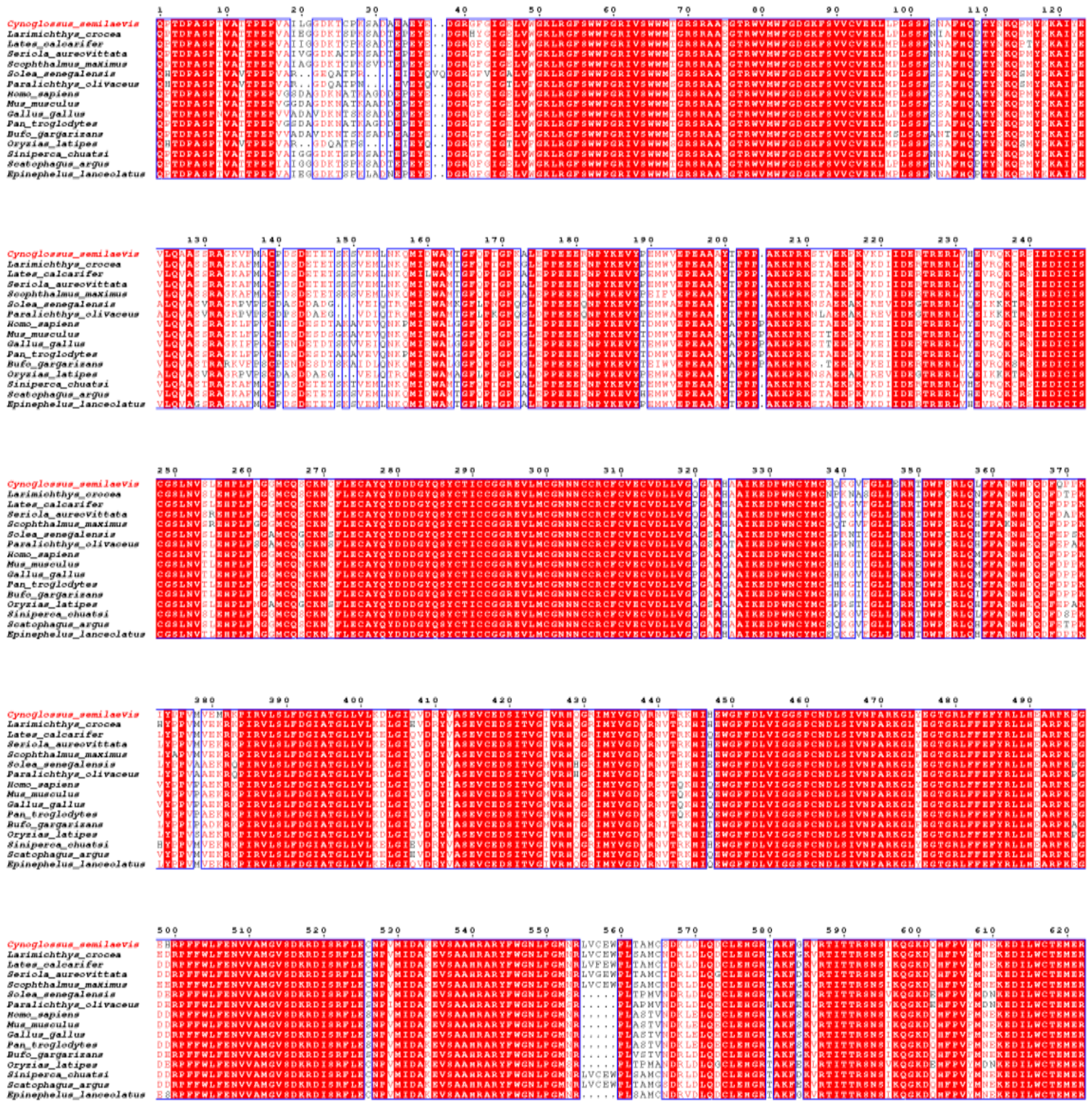

Figure S3. Sequence alignment of the dnmt3a protein from Chinese tongue sole with those of other vertebrate orthologues. Dark red shading identical residues. Chinese tongue sole is marked in red font.

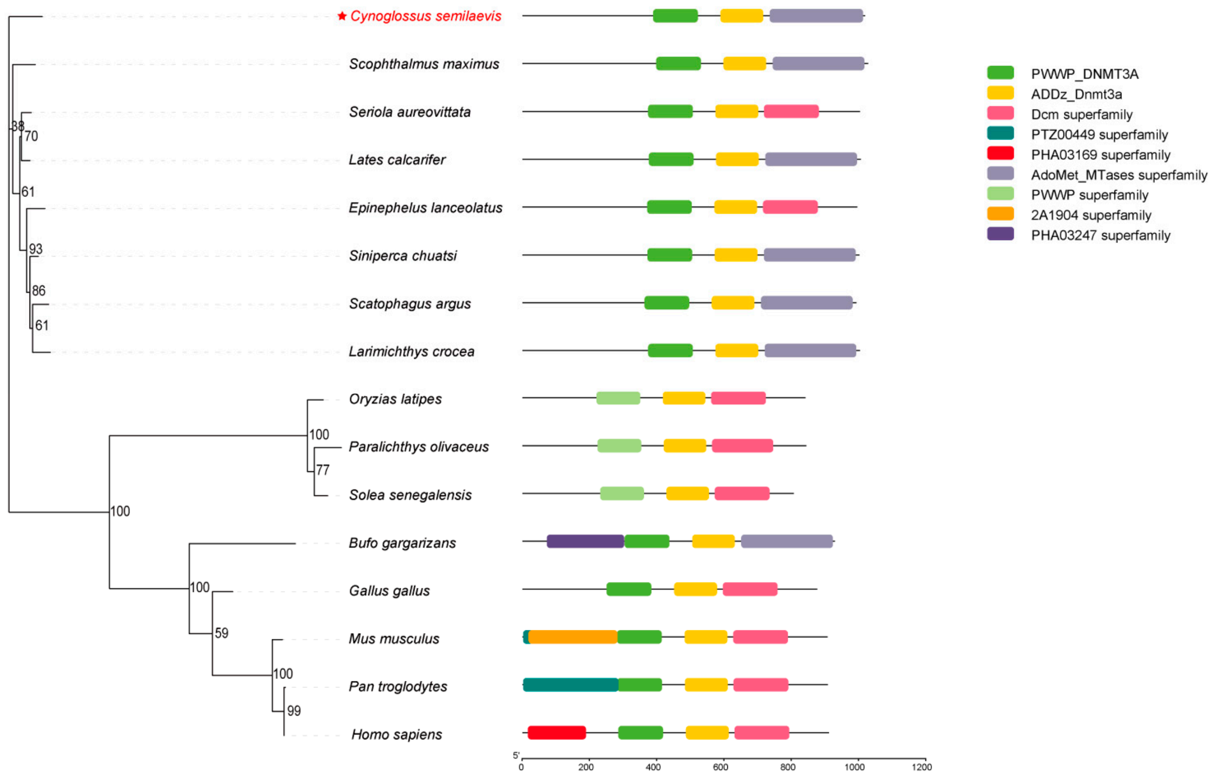

Figure S4. Phylogenetic analysis of dnmt3a proteins. The numbers at the branches of the phyligenetic tree stand for the bootstraps. Chinese tongue sole dnmt3a is coloured red. The domains corresponding to each species are showen on the right. The species marked by red stars is Chinese tongue sole (*Cynoglossus semilaevis*).

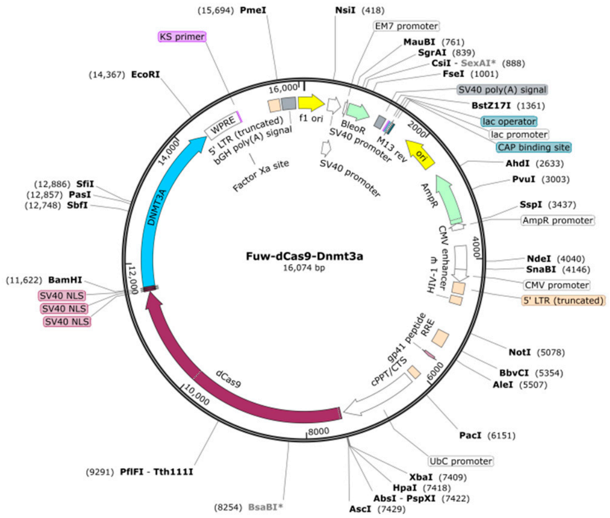

Figure S5. Plasmid profiles of dCas9-*dnmt3a*. Plasmid size is 16,398 bp and blue bar represents the *dnmt3a*. *, the site is blocked by Dcm methylation.

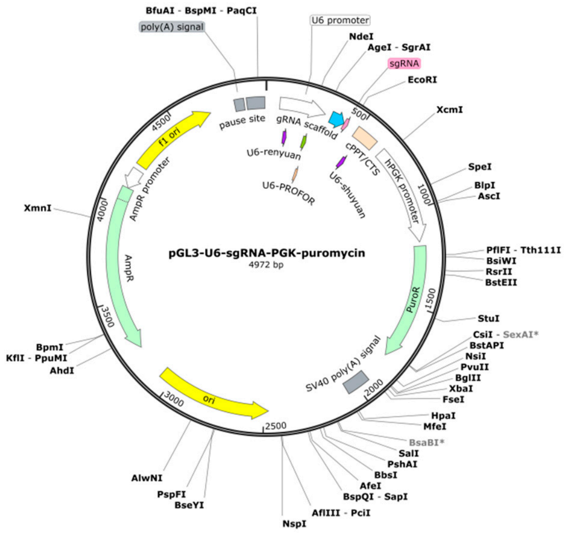

Figure S6. Plasmid profiles of pGL3-U6-sgRNA plasmid. Plasmid size is 4,973 bp and pink bar represents the genic position of *dnmt3a*. *, the site is blocked by Dcm methylation.

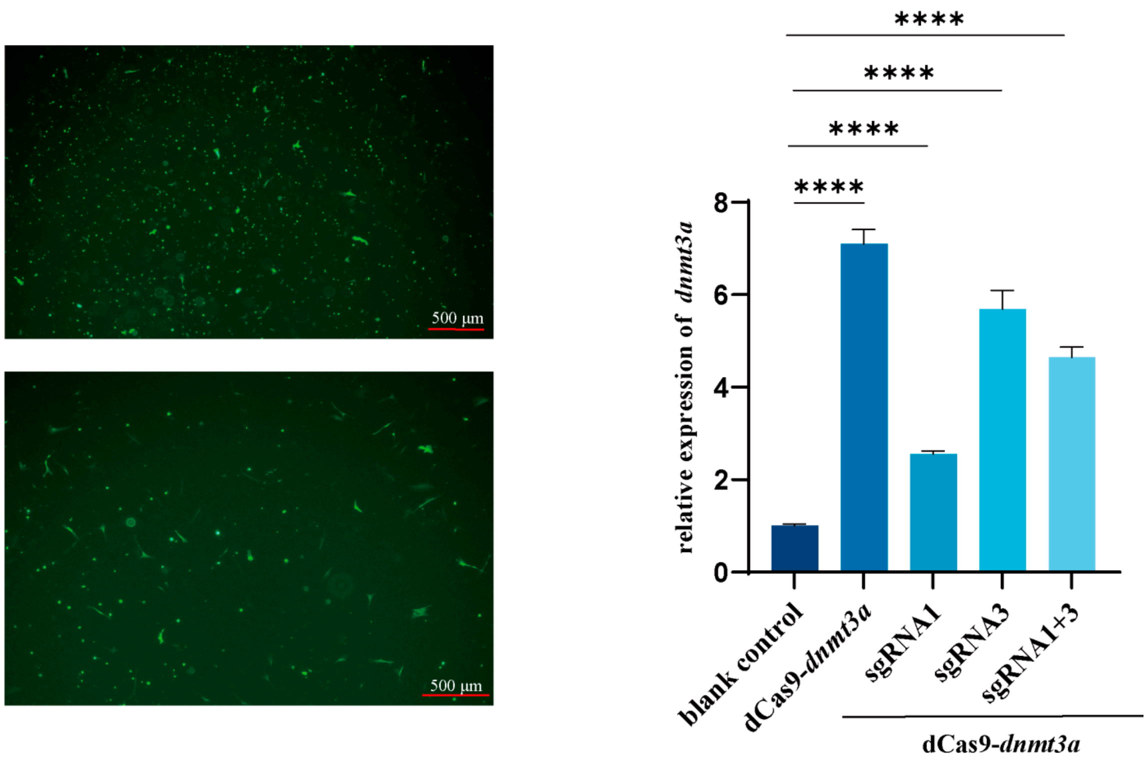

Figure S7 Transfection efficiency. The left panel shows GFP fluorescence. The panel on the right shows the qPCR of dnmt3a relative expression. Upper black lines indicate statistical difference among blank control and sgRNAs groups, while lines below the graph highlight the sgRNAs were co-transfected with the CRISPR/dCas9-*dnmt3a* vector. The asterisks (*) are used to denote significant differences among groups using Two-way ANOVA and Turkey's multiple comparisons. **** $p < 0.0001$.

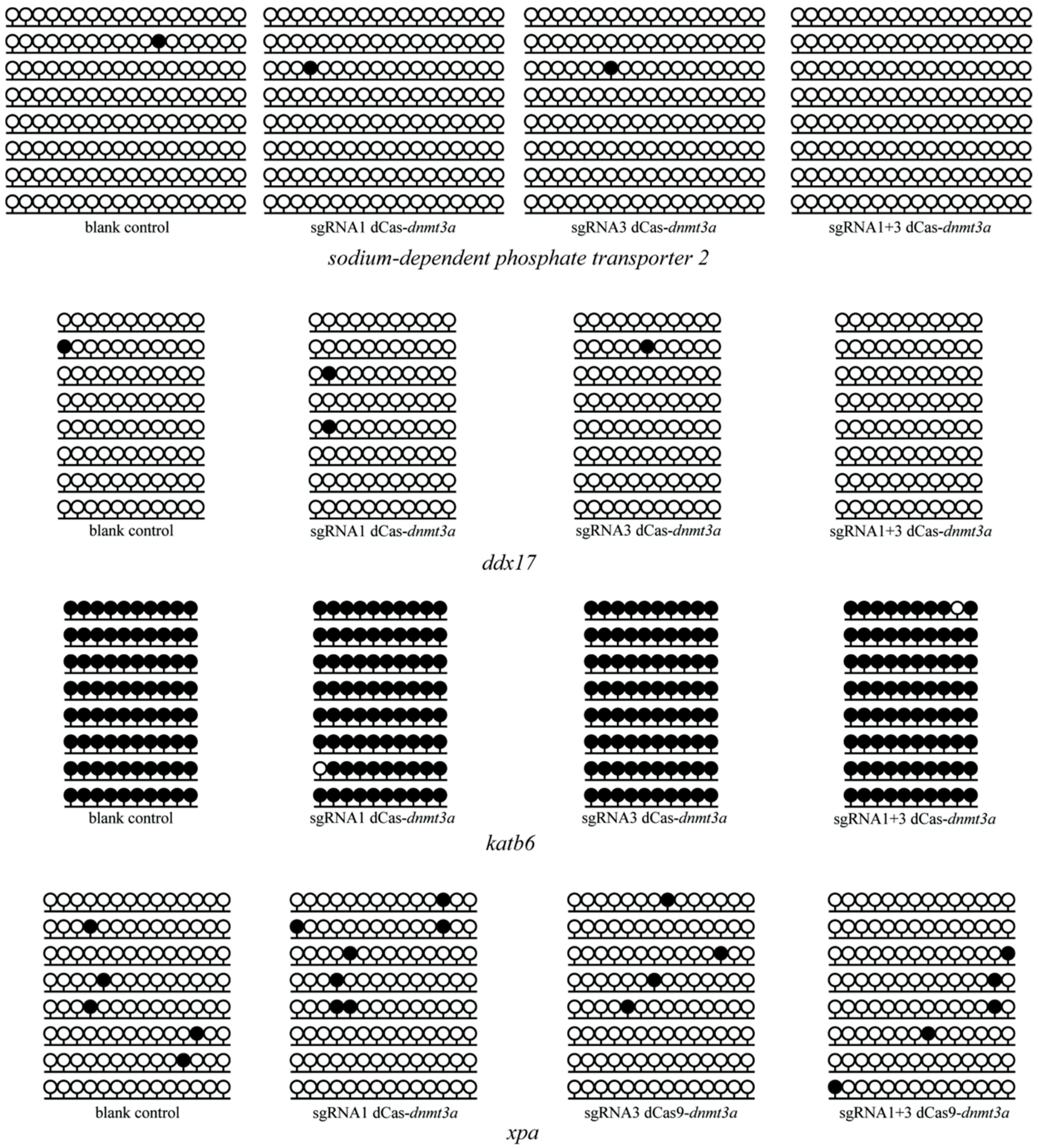

Figure S8. DNA methylation status of potential off-target genes. Gene names are given below the corresponding DNA methylation status profile. Filled (black) circles correspond to methylated Cs, unfilled (white) circles correspond to unmethylated Cs.

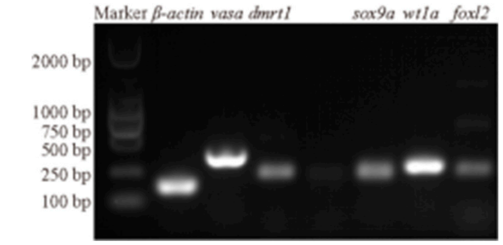

Figure S9. RT-PCR analysis of the gonadal somatic cell marker genes (*foxl2*, *sox9* and *wt1a*) and germ cell marker genes (*dmrt1 and vasa*). The RT-PCR results were confirmed in at least 3 batches of independent experiments, and representative results are shown.

Table S1. Amplification primers for *dnmt3a*

Primers	sequencing
dnmt3a-1-F	ATGATGCCGTCCAACGCCGT
dnmt3a-1-R	ACTTAGGGCAGGTCTTGTCTCC
dnmt3a-2-F	GACAAGGACGAAGACAGCCTG
dnmt3a-2-R	CCACGTAGCGGTCCACCTGA
dnmt3a-3-F	AGGATTTTCAACCCCCAAAG
dnmt3a-3-R	TCAAACACAGGCAAAGTATTCT

Table S2. Primers for sgRNAs plasmids construction.

Primers	sequencing
emx2-sgRNA1-F	accgGCGGACTCGAGCGCGCAACAgttttagagctagaaatagcaagttaaaa
emx2-sgRNA1-R	TGTTGCGCGCTCGAGTCCGCcggtgtcctttccacaagatatataaagcc
emx2-sgRNA2-F	accgCTCGCCTTAAGGTGGCGAAGgttttagagctagaaatagcaagttaaaa
emx2-sgRNA2-R	CTTCGCCACCTTAAGGCGAGcggtgtcctttccacaagatatataaagcc
emx2-sgRNA3-F	accgCGCGGTCCCTGTGCATCCCGgttttagagctagaaatagcaagttaaaa
emx2-sgRNA3-R	CGGGATGCACAGGGACCGCGcggtgtcctttccacaagatatataaagcc
emx2-sgRNA4-F	accgCCGGCGGCTTCGACTTAAACgttttagagctagaaatagcaagttaaaa
emx2-sgRNA4-F	GTTTAAGTCGAAGCCGCCGGcggtgtcctttccacaagatatataaagcc
emx2-sgRNA5-F	accgGCTCCTCGGAGCGGGACGAAgttttagagctagaaatagcaagttaaaa
emx2-sgRNA5-F	TTCGTCCCGCTCCGAGGAGCcggtgtcctttccacaagatatataaagcc
emx2-sgRNA6-F	accgCGCGAACACCAAGTCCGGGTgttttagagctagaaatagcaagttaaaa
emx2-sgRNA6-F	ACCCGGACTTGGTGTTCGCGcggtgtcctttccacaagatatataaagcc

Table S3. Primers for gene amplification in qPCR.

Primer	Sequencing
emx2-F	TGGGACACAGGTTCCAAGGTAA
emx2-R	TCCAACTTCTGCCGCTTGAA
β-actin-F	GCTGTGCTGTCCCTGTA
β-actin-R	GAGTAGCCACGCTCTGTC
sodium-dependent phosphate transporter 2-F	ATCTTTGCATGCCCGGATGA

sodium-dependent phosphate transporter 2-R	ACACCTTCCTTCACCAACGA
xpa-F	CCCTGTCGGTCTGTGACAAG
xpa-R	GGCCTCTTTTCTCCACCTGTA
ddx17-F	AATGAGGAGAGATGGGTGGC
ddx17-R	CCGTACGTCCGATACGATGG
katb6-F	AAAGGGGTCACCTGGCCTTG
katb6-R	AATAACGGCAGGACATCGCA
dnmt3a-F	CAGGTTGGTGTGTGAATGGC
dnmt3a-R	GGAAAACCGAAGATCCTCTCCA
wnt1-F	ATCAGTCACCGAGGGGATCA
wnt1-R	TCATGGTGCCTTCGGACAAG
klf-F	TCCATGCAGTACCAAGAGACG
klf-R	TTGACACTGAAACGGTCGGT
myc-F	CTGGAGAAGGTGGTCTCCGA
myc-R	GCAATGGGGTAGGGGAAGAC

Table S4. Primers of bisulfite sequencing PCR.

Primer	Sequencing
emx2-F	TTTTTTTGATTAAAGGGAATAAATTAAA
emx2-R	TCCCCATTTAATACTATTTAATAACCAC
sodium-dependent phosphate transporter 2-F	ATATATGTTTTGTGTTTATGTTTGG
sodium-dependent phosphate transporter 2-R	AACCTAACAATTCTATAATAAAATCC
xpa-F	GGTGAAAGAAATTTATTTTAAAGTTTT
xpa-R	TCTATCAAAACAATTTTACCTACCTTC
ddx17-F	TTGTAATAATAGAGTAATGATGTAATAGTG
ddx17-R	CCTAAAATAAATTTAATAAACAAATACTAA
katb6-F	TTAGATTGGGAGAGAATATAATATTATTTA
katb6-R	ACAAATTAAAATACAAAAAATCAAC
